# A double-blinded, randomized, placebo-controlled trial assessing the effects of nifedipine on embryo transfer: Study protocol

**DOI:** 10.1097/MD.0000000000009194

**Published:** 2017-12-22

**Authors:** Kelvin KL Ng, Genia Rozen, Tanya Stewart, Franca Agresta, Alex Polyakov

**Affiliations:** a Melbourne Medical School, University of Melbourne; b Department of Reproductive Services, Royal Women's Hospital, Parkville; c Melbourne IVF, East Melbourne, Victoria, Australia.

**Keywords:** contraction, embryo transfer, IVF, nifedipine, uterus

## Abstract

**Introduction:**

Implantation failure is the main factor affecting the success rate of in vitro fertilization (IVF) procedures. Studies have reported that uterine contractions (UCs) at the time of embryo transfer (ET) were inversely related to implantation and pregnancy rate, hence reducing the success of IVF treatment. Various pharmacological agents, with the exception of calcium channel blocker (CCB), have been investigated to reduce UC. In this regard, we are presenting a proposal for a double-blind randomized placebo-controlled trial. The trial aims to determine whether nifedipine, a CCB with potent smooth muscle relaxing activity and an excellent safety profile, can improve the outcome of ET.

**Methods and analyses:**

We will recruit 100 infertile women into one of 2 groups: placebo (n = 50) and nifedipine 20 mg (n = 50). Study participants will be admitted 30 minutes prior to ET and given either tablet after their baseline vital signs have been recorded. They will then undergo ET and be observed for adverse events for another 30 minutes post-ET. The primary outcome will be implantation rate and clinical pregnancy rate. Secondary outcomes include adverse events, miscarriage and pregnancy, and neonatal outcomes. Resulting data will then be analyzed using *t* test, Chi-square test, and multivariate test to compare outcomes between the 2 groups for any statistical significance. This protocol has been designed in accordance with the SPIRIT 2013 Guidelines.

**Key Points**

- Significant preliminary research has been performed to form the basis for the design of this trial.
- This is a randomized, placebo-controlled, double-blind trial to assess nifedipine's effect and safety at the time of embryo transfers.
- This study has the potential to provide a foundation for future large randomized controlled trials
- This trial is a well-designed randomized controlled trial to assess clinically relevant outcomes.
- Main limitation of this study is that uterine contractions are not measured and existing variability in embryo quality.

## 1 Introduction

Assisted reproduction technology (ART), from ovarian stimulation to the choice of catheters in embryo transfer (ET), has benefitted from major advances and improvements in recent years to continue to circumvent human infertility. However, implantation rate (IR) and clinical pregnancy rate (CPR) in women following ET have remained lower than desired. The IR and CPR are dependent on multiple factors such as techniques and materials involved in the ET process, quality of embryo, and also receptivity of the endometrium.^[1]^ Extensive research has been undertaken to understand and to improve the success of ET following in vitro fertilization (IVF)/intracytoplasmic sperm injection (ICSI) cycles.

One of the major limiting factors affecting the success of ET is thought to be the failure of embryo implantation in the uterus. Embryo implantation failure was estimated to be up to 85%^[2]^ and is governed by complex mechanisms depending on the quality of both the embryo and endometrium.^[3,4]^

Although embryonic aneuploidy is thought to be the major embryonic limiting factor for successful implantation,^[3]^ excessive uterine contractions (UCs) has been proposed as the endometrial limiting factor contributing to the reduced IR in IVF/ICSI cycles.^[3–5]^ As contractile or peristaltic activities of the uterus could move the implanted embryo toward the fallopian tube or cervix/vagina^[6]^ or might even expel it completely out of the uterus.^[3,4]^ This peristaltic mechanism toward the fallopian tube has been suggested to be the cause of higher ectopic pregnancy rate seen in ART.^[6,7]^ A recent published study demonstrated that the uterine peristaltic wave frequency before ET was inversely related to CPR in fresh and frozen–thawed cycles.^[7]^

Various pharmacological agents have been investigated to reduce UC since. These include cyclo-oxygenase inhibitors, β2 adrenoreceptor agonists, antiinflammatories, phosphodiesterase inhibitors, progesterone, and antispasmodics^[8–23]^ demonstrating variable results. Atosiban, an oxytocin/vasopressin receptor antagonist, has shown to be the most promising in improving IR and CPR.^[13]^

If excessive UC indeed reduces IR and CPR in women undergoing IVF/ICSI cycles, then it represents a potential target for pharmacological agents to improve the success of IVF/ICSI cycles.

Calcium channel blockers (CCBs) are nonspecific smooth muscle relaxants, predominantly used for the treatment of hypertension in adults. It specifically inhibits the transmembrane calcium influx at the voltage-gated L-type channels.^[24]^ Since these channels are expressed widely throughout body, including the myocardial cells, vasculature smooth muscles, and the myometrium, CCB exhibits multiorgan effects.^[25]^ By inhibiting the slow inward current of the action potential via reducing the intracellular levels of calcium,^[26]^ it decreases the contractility of the smooth muscles and hence causes vasodilation, uterine relaxation, and other effects throughout the body.^[24]^

The most widely used and studied CCB is nifedipine, and there is evidence for safety in pregnancy.^[27–31]^ Nifedipine belongs to a subclass of CCB, namely dihydropyridine, which is selective to vascular over cardiac tissues of about 10:1.^[27]^ Nifedipine, as a CCB, has vasodilatory and potent uterine relaxation properties.^[24]^ It appears that nifedipine is a very safe drug with limited major adverse effects and has demonstrated superiority over other drugs, such as labetolol and β2 adrenoreceptor agonists, in the management of preterm labour or pregnancy induced hypertension consistently.^[24,27–29,32–37]^

As nifedipine is an antihypertensive and has cardiodepressive effect,^[38]^ patients who suffer from hypotensive or compromised cardiovascular conditions are contraindicated to the use of nifedipine.^[34]^ For others, the most common maternal side effects, when nifedipine is used in a therapeutic range, are: transient facial flushing, headache, nausea, tachycardia, and hypotension; less common side effects are: palpitations, dizziness, chest pain, nasal congestion, oedema, and heartburn.^[25,28,37,39–40]^ These side effects are mostly benign and are resolved spontaneously or by withdrawal of administration.^[37,39–40]^ Naturally, adverse events vary according to the total dose of nifedipine^[28]^ and these data arise from studies based on multiple dosing regimen but no data are available for a single dose regimen. Current recommended maximum dosage is 120 mg per day^[31]^ but maximum dose of 160 mg per day has been reported to achieve tocolysis with no increase in maternal side effects in 1 study.^[41]^ Nevertheless, caution should be exercised when prescribing any medication in any clinical scenario.

There is currently no evidence to demonstrate nifedipine toxicity on human embryos.^[42–46]^ More importantly, a long-term study following 94 neonates for up to 18 months of life has found no impact on malformations, diseases incidences, motor function, or childhood education following nifedipine exposure in utero^[47]^ while another large case–control study also found no increased in congenital anomalies risk.^[48]^ Improved neonatal outcomes, such as neonatal morbidity, have also been demonstrated over other tocolytics and antihypertensives in the treatment of pregnancy induced hypertension and preterm labor.^[28,32,36,49,50–56]^

## 2 Rationale

Implantation failure remains a major factor affecting the success rate of IVF treatment. There is enough evidence to demonstrate that excessive uterine activities are detrimental to the outcome of ET. Given nifedipine's potent uterine relaxing properties and excellent safety profile, it is a promising candidate to improve IVF outcomes. There is a dearth of information in the literature regarding the use of nifedipine in ART. Therefore, the objective of this study is to evaluate the efficacy of nifedipine administration in improving implantation and pregnancy rates in IVF/ICSI fresh and frozen–thawed ETs.

## 3 AIMS

We aim to establish the clinical benefit and other outcomes of ET from a single-dose nifedipine administration as a smooth muscle relaxant.

Specific outcomes are:

(1) IR
(2) CPR
(3) Adverse events rate, including miscarriages
(4) Live birth rate
(5) Pregnancy and neonatal outcomes

## 4 Methods and analyses

This protocol has been designed in accordance with the SPIRIT 2013 Guidelines. Recruitment will start from the end of 2016 for approximately 12 months. Analysis and dissemination will occur after this period of time. Analysis will be by intention to treat and the study is to expect to be completed by the beginning of 2018, approximately the 1st of February 2018, with all live births and secondary outcomes data analyzed.

## 5 Study design

Interventional phase I double-blinded randomized placebo-controlled trial.

## 6 Participants

We plan to recruit 100 infertile women undergoing ETs in IVF/ICSI cycles.

## 7 Study setting

Patients will be recruited from Melbourne IVF over a period of 12 months. This should allow enough time to achieve adequate participant enrolment to reach target sample size.

## 8 Interventions to be measured

This trial will have 2 arms. All capsules will be indistinguishable from each other.

(1) Placebo tablet taken once, 30 minutes pre-ET.
(2) 20 mg immediate release nifedipine tablet taken once, 30 minutes pre-ET

## 9 Primary outcome

(1) IR, defined as the presence of serum human chorionic gonadotropic level >5 IU/L following ET.
(2) CPR, defined as the presence of a live pregnancy in the uterine cavity at a transvaginal ultrasound at 6 weeks’ gestation onwards.

## 10 Secondary outcome

(1) Adverse events defined as any unwanted outcomes, such as dizziness or hypotension, reported in this trial.
(2) Miscarriage rate defined as loss of a diagnosed clinical pregnancy before 20 weeks gestation.
(3) Pregnancy and neonatal outcomes including multiple pregnancy, congenital or chromosomal abnormalities, stillbirth, preeclampsia, delivery before 34 weeks, delivery between 34 and 37 weeks, necrotising enterocolitis, abnormal neurology, placenta praevia, gestational diabetes, low birth weight, admission to neonatal intensive care unit, duration of admission, need and duration of respiratory support, or any other neonatal morbidity reported.

## 11 Sample size

As this is a small scale study, without precedence on which to base accurate power calculations, a power calculation has not been performed. This study should provide guidance for future larger randomized trials. We hypothesis that nifedipine will improve the IR and CPR after IVF/ICSI cycles. A convenient sample of 100 participants, 50 in each arm, was chosen to assess our primary and secondary outcomes.

## 12 Inclusion criteria

(1) A total of 18–45 year females undergoing IVF/ICSI cycles and fresh or frozen–thawed ET.
(2) Baseline BP greater than or equal to 100/60 mm Hg measured pre-ET.

## 13 Exclusion criteria

(1) Body mass index (BMI) greater than 38.
(2) Early follicular phase (day 2–4) serum follicle-stimulating hormone level >20 mIU/mL.
(3) Abnormal uterine cavity as evidenced by sonohysterogram or hysterosalpingography.
(4) Any contraindication to being pregnant and carrying a pregnancy to term.
(5) Contraindication for the use of nifedipine, estrogen, and progesterone suppositories.
(6) Patient being treated with other drugs that interact with cytochrome P450 activity: azole antifungals, cimetidine, cyclosporine, erythromycin, quinidine, terfenadine, warfarin, benzodiazepines, flecainide, imipramine, propafenone, and theophylline.
(7) Irregular heart beat or already being treated with another medication for high blood pressure.
(8) Any ovarian or abdominal abnormality that may interfere with adequate transvaginal sonography (TVS) evaluation.
(9) Administration of any investigational drugs within 3 months prior to study enrolment.
(10) Patient not able to communicate adequately with the investigators and to comply with the requirements of the entire study.
(11) Unwillingness to give written informed consent.
(12) Previous entry into this study.
(13) Baseline BP less than 100/60 mm Hg measured pre-ET.
(14) Embryos that have undergone preimplantation genetic screening

## 14 Concomitant care

Aside from trial protocol and inclusion criteria listed above, standard concomitant care, such as folic acid supplements, is permitted.

## 15 Participant enrolment

This trial will begin recruitment at the level of infertility specialist. Once identified as requiring IVF/ICSI, and satisfying inclusion and criteria, the patient will be provided with detailed written information about the trial protocol. Once informed consent is obtained, then the patient will be randomized to one of the study arms and provided with the trial medication 30 minutes prior to their scheduled ET.

## 16 Allocation concealment, blinding, and randomization

Randomization will be performed, by a research assistance, via a computer generated sequence in blocks of 10 recruited subjects. The allocation ratio into each arm will be 1:1. The randomization will be stratified for age into 2 groups: less than 35 years old and greater or equal to 35 years old. Neither patients nor caregivers will be aware of the allocation.

All clinical staff will remain blinded to allocation of therapy until the statistical data base is cleaned and locked. Unblinding only be done in case of urgent medical need.

## 17 Adherence and retention

To ensure the integrity of study data, participants adherence to trial protocol, that is, nifedipine administration, will be supervised by the clinical nurse. The time of drug administration, blood pressure pre- and post-ET, and the time of ET will be recorded on the patient record form. Every reasonable effort will be made from the time of enrolment until the end of follow-up to maintain contact with and maintain participant's participation in the trial.

## 18 Analysis plan

Primary analysis will occur by the intention-to-treat principle. Statistical comparisons would be carried out using Chi-square test, Fisher exact test, and Student *t* test with the Statistical Program for Social Science (SPSS, Inc., Version 23.0, Chicago, IL). A 2-sided *P* < .05 was taken as statistically significant. Primary and secondary outcomes between placebo group and treatment group would be analyzed using the above statistical comparisons. The baseline demographics of participants, such as age and number of embryo transferred, would also be analyzed to ensure adequate randomization.

## 19 Adverse events and data safety and monitoring

During the treatment period, clinical care will remain unchanged and will be the responsibility of uninvolved medical staff. The principal investigators will be available by telephone at all times during the trial, and participants will be provided with contact details in case of any adverse events. Participants will also be screened at the time of their clinical visits, including ultrasound and oocyte collection appointments prior ET to ensure that patients are fit to participate in the research. Blood pressure and heart rate would be measured 30 minutes pre- and post-ET. If baseline blood pressure pre-ET is less than 100/60 mm Hg, participation in the study will not be allowed for safety purposes. Adverse events and side effects are recorded 30 minutes post-ET. Serious adverse events will be recorded separately and followed up until resolution. Such events will be reported to the principle investigators, Melbourne IVF research committee, Melbourne IVF Human Research Ethics Committee, Melbourne IVF quality management system, indemnity insurer, and if directed by HREC, to the Australian Therapeutics and Goods Administration.

## 20 Trial modification and discontinuation

In the absence of adverse events, the medication regimen will not be modified once started. If other protocol changes are deemed necessary by the investigating team, ethical approval will be sought from approving Human Research Ethics Committee. Once approved, protocol amendments will be notified to all investigators, administrators, and trial participants.

Patients are permitted to withdraw from study participation at any time. The trial will cease follow-up once patients have given birth or when clinical pregnancy is not detected from the trial treatment cycle.

## 21 Data collection, informed consent forms, and confidentiality

Data will be recorded in hardcopy and electronic form. Hardcopies will be stored in a secured filing cabinet at the administering institution. Electronic copies will be stored on a password protected computer. Final trial dataset would only be accessible for this study's clinical investigators after recruitment and data collection has been completed. All data will be kept for 15 years; following this time, hardcopies will be destroyed by shredding or burning and electronic copies will be deleted by formatting. Participant records will not contain any directly identifiable information and no biological samples would be collected.

## 22 Ethics and dissemination

Data analysis, interpretation, and conclusion will be presented at national and international conferences and published in peer-reviewed journals.

## 23 Discussion

Implantation failure is a major factor limiting the success of IVF/ICSI treatment and excessive UCs is one of the mechanisms contributing to implantation failure. Given nifedipine's tocolytic properties, safety profile, and use in obstetrics practice, it is a promising candidate to improve the outcomes of IVF/ICSI treatment. This trial is essential to confirm the efficacy, side effects, and safety of nifedipine in infertility treatment. If this study confirms that nifedipine favors a positive outcome, we will then proceed to a phase II randomized controlled trial. A phase I trial to investigate the clinical benefits, namely improved implantation and pregnancy rate of nifedipine in IVF/ICSI treatment cycle, has been initiated.
